# Symptoms of anxiety/depression during the COVID-19 pandemic and associated lockdown in the community: longitudinal data from the TEMPO cohort in France

**DOI:** 10.1186/s12888-021-03383-z

**Published:** 2021-07-28

**Authors:** Astrid Juhl Andersen, Murielle Mary-Krause, Joel José Herranz Bustamante, Mégane Héron, Tarik El Aarbaoui, Maria Melchior

**Affiliations:** Sorbonne Université, INSERM, Institut Pierre Louis d’Épidémiologie et de Santé Publique (IPLESP), Equipe de Recherche en Epidémiologie Sociale (ERES), F75012 Paris, France

**Keywords:** Mental health, Symptoms of anxiety/depression, Lockdown, Longitudinal study, France

## Abstract

**Background:**

To cope with the COVID-19 pandemic, social distancing restrictions where implemented in France, which could have led to social isolation. This is expected to have affected the mental health situation, including increasing risk of symptoms of anxiety and depression in the general population. Persons with prior mental health difficulties could be an especially vulnerable group, however, few studies have tested this empirically considering preexisting mental health difficulties. We examine the association between preexisting symptoms of anxiety/depression and anxiety/depression during lockdown due to the COVID-19 pandemic in a longitudinal community sample.

**Methods:**

A longitudinal follow-up during lockdown (data collection March–June 2020) was implemented among participants of the TEMPO cohort. Prior knowledge of anxiety/depression was included from prior waves of data collection. Generalized estimation equations models were used to estimate the association between preexisting symptoms of anxiety/depression and symptoms of anxiety/depression during lockdown among 662 mid-aged individuals.

**Results:**

Individuals with symptoms of anxiety/depression measured prior to lockdown had 6.73 higher odds [95% CI = 4.45–10.17] of symptoms of anxiety/depression during lockdown. Additionally, the likelihood of symptoms of anxiety/depression during lockdown was elevated among women (OR = 2.07 [95% CI = 1.32–3.25]), subjects with low household income (OR = 2.28 [1.29–4.01]) and persons who reported being lonely (OR = 3.94 [95% CI = 2.47–6.28]).

**Conclusions:**

Our study underlines the role of preexisting symptoms of anxiety/depression as a vulnerability factor of anxiety/depression during lockdown. Interventions focusing on individuals with mental health difficulties as well as people feeling lonely should be considered, to reduce the psychological impact of the COVID-19 pandemic.

**Supplementary Information:**

The online version contains supplementary material available at 10.1186/s12888-021-03383-z.

## Background

Since December 2019, the COVID-19 pandemic, caused by the severe acute respiratory syndrome coronavirus 2 (SARS-CoV-2), and the associated governmental restrictions have impacted daily life in most parts of the world. In France, the government introduced nationwide lockdown and home confinement on March 17, 2020 until May 11, 2020 [1], which stipulated severe restrictions on social contacts, on many people’s ability to work, and greatly reduced access to services.

Recent cross-sectional studies have estimated the prevalence of anxiety and depression symptoms during the COVID-19 pandemic in different non-French populations, [2–8] all finding high prevalence of generalized anxiety disorder (GAD) and depressive symptoms after the introduction of the pandemic. Since the COVID-19 outbreak, French adults seem to have an especially high prevalence of mental distress, including stress and anxiety, compared to other OECD countries [9]. Especially people feeling lonely, women, young people, those with preschool aged children, having a low income, or living in cramped housing are identified as especially vulnerable in terms of mental health in the context of the COVID-19 pandemic [4, 10–13]. A recent review has shown that quarantine during previous epidemics had a strong impact on mental health [14], and studies conducted in the context of other infectious disease outbreaks have reported that symptoms of anxiety and depression were elevated during the time of the event and several months after [15–17]. A deterioration in mental health outcomes is expected in the general population during a pandemic and lockdown period [18], but little is known about the impact of these special circumstances on persons with mental health difficulties before lockdown. Yet individuals with preexisting mental health disorders may be one of the particularly vulnerable groups, when it comes to the effects of the lockdown on population mental health [10, 12, 19, 20]. A systematic review investigating the COVID-19 pandemic and mental health, finds that patients with preexisting psychiatric disorders reported worsening in symptoms at the time of lockdown [20].

As in other countries, lockdown in France was associated with an injunction on physical distancing, where it was prohibited to meet with people from other households [1]. During the time of lockdown, loneliness had been considered as an important concern for mental health [21], where loneliness was seen as the main risk factor for depression, anxiety and their comorbidity [22]. Even in times without lockdown, loneliness and mental health difficulties are found to be highly associated [10, 23, 24], while social isolation due to lockdown can be expected to have an impact on the risk of experiencing symptoms of depression and anxiety during lockdown [4, 25].

With the knowledge we have about the mental health situation during lockdown, and groups that appear especially vulnerable, there is reason to believe that persons who experienced symptoms of anxiety/depression before the COVID-19 pandemic are more vulnerable to internalizing symptoms during lockdown than those with no such symptoms experienced earlier. In line with this, the aim of this study is to examine the association between preexisting symptoms of anxiety/depression and symptoms of anxiety/depression during the COVID-19 outbreak among a mid-aged French population. Furthermore, we are interested in examining other factors related to symptoms of anxiety/depression during the first lockdown in France, including sex, household income and loneliness.

## Methods

### Study population and procedure

This study includes 662 participants from the TEMPO COVID-19 project, a longitudinal follow-up study aiming to better understand French adults’ mental health situation during the pandemic. Data was collected seven times starting on the 24th of March 2020, 1 week after France declared lockdown. The TEMPO COVID-19 project is nested within the TEMPO (Trajectoires ÉpidéMiologiques en POpulation) cohort, which has been described in detail elsewhere [26, 27]. Briefly the TEMPO cohort was set up in 2009 among young adults (22–35 years), who had previously taken part in a study on children’s psychological problems and access to mental health care in 1991 and 1999 [28, 29]. In 2009, participants from 1991 were contacted to complete the TEMPO study, and were followed via self-completed questionnaires in 2011, 2015 and 2018 [30]. From March to June 2020, TEMPO COVID-19 data were collected in seven periods of time. Invitations and reminders to participate were sent by email to all TEMPO participants with a valid email address, corresponding to a total of 1224 participants contacted at every time. Follow-up questionnaires were sent weekly for the first five data collections, and biweekly for data collections six and seven (for specific dates and number of participants see Supplementary Table 1).

### Measures

#### Outcome: anxious/depressive symptoms during lockdown

Mental health during lockdown was measured using items from the *Anxious/Depressed* syndrome scale based on the Adult Self Report (ASR)-Achenbach System. The ASR is a well-validated instrument to asses adult psychopathology and is used for both clinical and research purposes and includes in total 18 items [31, 32]. Each item is rated on a three-point scale from 0 (*Not true*), 1 (*Somewhat true or Sometimes*) and 2 (*Very Often or Often True*). To calculate scores of symptoms of anxiety and depression during lockdown, this study includes 8 items in the first follow-up questionnaire in 2020 and 13 items in the questionnaire used for the 2–7 follow up selected from the Anxious/Depressed syndrome scale [33] (for specific questions selected see Supplementary Table 2). In the present study the Anxious/Depressed syndrome scale was dichotomized. To create a comparable anxiety/depression measure across points of follow-up the scores were transformed to a 0–100 scale. According to ASR guidelines, the 85th percentile serves to identify persons with clinically significant symptoms [32]. The values on the 85th percentile for each data collection during COVID-19 were calculated, and the mean of these values resulted in a cut-off corresponding to a score of 34. This score was used to create a comparable dichotomous measure across the seven questionnaires during lockdown.

#### Preexisting symptoms of anxiety/depression

Information on participants’ past symptoms of anxiety and depression was included using the last available measure from prior waves 2018 (87%), 2011 (11%) or 2009 (2%) of the TEMPO cohort. In 2018 participants completed the same items from the ASR Anxious/Depressed syndrome scale as in TEMPO COVID-19 wave 1. For the subgroup that did not complete the 2018 TEMPO study questionnaire, mental health information was used from 2011, where participants reported information on major depression and generalized anxiety disorder subscales of the Mini-International Neuropsychiatric Interview (MINI) [34, 35]. For the remaining subjects with no information on anxiety/depression symptoms from 2018 or 2011, information on anxiety/depression were included from 2009, where participants also answered items from the ASR. If no information were available from those 3 years, subjects were excluded from the analysis.

#### Covariates

Participants’ demographic, occupational, and economic characteristics were collected in the first questionnaire answered during lockdown. Marital status was grouped into ‘single, divorced or widowed’ vs. ‘married or in a civil union.’ Living situation was coded as ‘living with a partner with children,’ ‘living with a partner without children’ and ‘other,’ and housing situation as ‘living in a house’ vs. ‘living in an apartment.’ Participants’ educational attainment was dichotomized based on whether individuals had obtained up to an initial university degree (2 years post high school or less) or a bachelor’s degree or higher (at least 3 years post high school). Furthermore, we identified whether participants lived in an area with a high level of COVID-19 infections in March to June 2020, where ‘yes’ included the Paris region or Eastern France and ‘no’ included rest on France [36]. Participants’ occupational status was defined by their employment type, which was divided into four groups; ‘stable’ defined as permanent contract, civil servant, or self-employed, ‘unstable’ defined as temporary contract or other short-term employment, ‘unemployed and looking for a job,’ and ‘unemployed and not looking for a job.’ Self-reported household income was dichotomized based on the best fitting category according to the median income in France (2500 euros or less per month vs. 2501 euros or more per month) [37]. Preexisting financial difficulties were self-reported in 2018 and covering basic necessities (rent, heating or electricity costs, medical care or medication, and eating varied, sufficient and balanced meals). For individuals who did not respond in 2018, we used information on financial difficulties in the 12 months preceding lockdown. In each questionnaire, participants were asked the UCLA (University of California, Los Angeles) 3-item Loneliness Scale [38], where the questions are rated from 1 (*Hardly never*) to 3 (*Often*). Individuals with a summed score from 3 to 5.9 were considered as “not being lonely,” and those with a score from 6 to 9 as “lonely” [39].

### Statistical analyses

To study the association between symptoms of anxiety/depression during lockdown and the previous measure of anxiety/depression, we started describing the TEMPO COVID-19 sample participants’ characteristics and their preexisting mental health situation. Second, we implemented bivariate and multivariate generalized estimation equation (GEE) models, with a logit link and binomial distribution as well as an unstructured correlation matrix, to examine the relationship between the outcome, that is, anxious/depressive symptoms during lockdown, and previous measure of anxiety/depression symptoms and covariates. To control for potential confounders, all covariates with a *p*-value < .20 in the bivariate GEE model was included. Factors adjusted for in the final model were: sex, living situation, occupational status, household income and measures of loneliness during lockdown. Lastly, interactions between previous measured symptoms of anxiety/depression and covariates were tested. Statistically significant interactions were included in the final model. All analyses were performed using SAS (version 9.4), and are based on a complete case analysis.

## Results

In total, 729 individuals participated in at least one of the seven follow-up questionnaires during lockdown, 67 participants were excluded due to missing information, yielding a total sample of 662 subjects with complete data on at least one of the seven study questionnaires. In most cases (87% [*n* = 575]) information on preexisting mental health difficulties was available in the 2018 TEMPO wave, and for subgroups in 2011 (11% [*n* = 76]) and in 2009 (2% [*n* = 11]). Descriptive statistics are presented in Table 1.

**Table 1:** Socio-demographic characteristics of TEMPO cohort study participants and mental health status prior to COVID-19-related lockdown (March–June 2020)

	Total	Preexisting symptoms of Anxiety/Depression^c^		
		No	Yes	*p*-value
	662 (100%)	495 (74.8%)	167 (25.2%)	
Sex^a^				
Male	237 (35.8%)	204 (41.2%)	33 (19.8%)	< 0.0001
Female	425 (64.2%)	291 (58.8%)	134 (80.2%)	
Age^a^				
Mean (SD)	39.8 (3.65)	39.9 (3.70)	39.3 (3.47)	0.3252
Marital status^a^				
Married, or in a civil union	528 (79.8%)	401 (81.0%)	127 (76.0%)	0.1676
Single, divorced or widowed	134 (20.8%)	94 (19.0%)	40 (24.0%)	
Living situation^a^				
Living with partner and children	434 (65.6%)	343 (69.3%)	91 (54.5%)	0.0016
Living with partner without children	70 (10.6%)	44 (8.9%)	26 (15.6%)	
Other	158 (23.9%)	108 (21.8%)	50 (29.9%)	
Housing situation^a^				
In a house	450 (68.0%)	343 (69.3%)	107 (64.1%)	0.2111
In an apartment	212 (32.0%)	152 (30.7%)	60 (35.9%)	
Educational attainment^a^				
Bac + 3 or higher	471 (71.2%)	355 (71.7%)	116 (69.5%)	0.5779
Bac + 2 or lower	191 (28.8%)	140 (28.3%)	51 (30.5%)	
Living area^a^				
Low level of COVID-19 incidence	468 (70.7%)	352 (71.1%)	116 (69.5%)	0.6854
High level of COVID-19 incidence	194 (29.3%)	143 (28.9%)	51 (30.5%)	
Occupational status^a^				
Stable contract type	593 (89.6%)	451 (91.1%)	142 (85.0%)	0.1235
Unstable contract type	31 (4.7%)	20 (4.1%)	11 (6.6%)	
Not working, but looking for a job	24 (3.6%)	14 (2.8%)	10 (6.0%)	
Not working, not looking for a job	14 (2.1%)	10 (2.0%)	4 (2.4%)	
Household income^a^				
2501 euros or more	542 (81.9%)	421 (85.1%)	121 (72.5%)	0.0003
2500 euros or less	120 (18.1%)	74 (14.9%)	46 (27.5%)	
Financial difficulties^b^				
No	587 (88.7%)	449 (90.7%)	138 (82.6%)	0.0044
Yes	75 (11.3%)	46 (9.3%)	29 (17.4%)	
Loneliness^d^				
No	568 (85.8%)	440 (88.9%)	128 (76.6%)	< 0.0001
Yes	94 (14.2%)	55 (11.1%)	39 (23.4%)	

^a^Measured each time a participant answered to TEMPO COVID-19 questionnaires for the first time.

^b^Measured 2018 [*n* = 566], or if missing in first wave answered in TEMPO COVID-19 (past 12 month) [*n* = 96].

^c^Measured in 2018 [*n* = 575], or if missing in 2011 [*n* = 76] or 2009 [*n* = 11].

^d^Measured each time a participant answered to TEMPO COVID-19 questionnaires, and average score was calculated.

Females represented 64% of study population. Participants were on average 40 years old, a majority were married, living with a partner and children, and working in stable work contract. Furthermore, most participants lived in a house and in a low COVID-19-incidence area (29% were living in the Paris region or Eastern France, where the incidence of COVID-19 was highest in March–June 2020).

Table 2 presents the results of bivariate GEE models, showing that preexisting symptoms of anxiety/depression were associated with significantly higher odds of symptoms of anxiety/depression during lockdown.

**Table 2:** Preexisting symptoms of anxiety/depression and covariates in relation to symptoms of anxiety/depression during COVID-19 related lockdown (March–June 2020). Bivariate GEE regression models (Unadjusted Odds Ratios (OR) and 95% Confidence Interval (CI), TEMPO cohort study, *n* = 662)

Symptoms of Anxiety/Depression during COVID-19 lockdown			
	OR	95% CI	*p*-value
Preexisting symptoms of Anxiety/Depression at least once			
No	1		
Yes	6.74	[4.71–9.65]	< 0.0001
Sex			
Male	1		
Female	2.59	[1.71–3.93]	< 0.0001
Age	0.97	[0.93–1.02]	0.1934
Living situation			
Living with partner and children	1		
Living with partner without children	1.51	[0.84–2.73]	0.1683
Other	1.45	[0.84–2.73]	0.0630
Housing situation			
In a house	1		
In an apartment	0.84	[0.57–1.22]	0.3554
Educational attainment			
Bac + 3 or higher	1		
Bac + 2 or lower	1.28	[0.88–1.86]	0.1979
Living area			
Low level of COVID-19 incidence	1		
High level of COVID-19 incidence	0.78	[0.54–1.17]	0.2531
Occupational status			
Stable contract type	1		
Unstable contract type	0.97	[0.44–2.14]	0.9475
Not working, but looking for a job	3.56	[1.74–7.29]	0.0005
Not working, not looking for a job	1.20	[0.37–3.83]	0.7635
Household income			
2501 euros or more	1		
2500 euros or less	2.67	[1.79–3.99]	< 0.0001
Loneliness			
No	1		
Yes	2.45	[1.91–3.13]	< 0.0001

Results of the multivariate adjusted model are shown in Table 3.

**Table 3:** Association between preexisting symptoms of anxiety/depression and symptoms of anxiety/depression during the COVID-19 related lockdown (March–June 2020). Multivariate GEE regression model (adjusted Odds-ratios (OR), 95% Confidence Interval (CI), TEMPO cohort study, *n* = 662)

Symptoms of Anxiety/Depression during COVID-19 lockdown			
	OR	95% CI	*p*-value
Preexisting symptoms of Anxiety/Depression			
No	1		
Yes	6.73	[4.45–10.17]	< 0.0001
Sex			
Male	1		
Female	2.07	[1.32–3.25**]**	0.0015
Living situation			
Living with partner and children	1		
Living with partner without children	0.73	[0.39–1.36]	0.3208
Other	0.48	[0.22–1.06]	0.0689
Occupational status			
Stable contract type	1		
Unstable contract type	0.58	[0.23–1.46]	0.2441
Not working, but looking for a job	1.92	[0.89–4.16]	0.0972
Not working, not looking for a job	0.85	[0.29–2.52]	0.7714
Household income			
2501 euros or more	1		
2500 euros or less	2.28	[1.29–4.01]	0.0044
Loneliness			
No	1		
Yes	3.94	[2.47–6.28]	< 0.0001

As hypothesized, a statistically significant association was found between preexisting symptoms of anxiety/depression and such symptoms during lockdown in March–May 2020 in the multivariate GEE analysis. Participants presenting symptoms of anxiety/depression prior to lockdown were about 7 times more likely to report symptoms of anxiety/depression during lockdown. Furthermore, being female, having a low household income and suffering from loneliness were associated with higher odds for symptoms of anxiety/depression during lockdown. A statistically significant interaction was identified between preexisting anxiety/depression and loneliness during lockdown (*p* = 0.04).

## Discussion

### Summary of findings

The aim of this study is to demonstrate the relationship between prior measured symptoms of anxiety/depression and anxiety/depression during the COVID-19 outbreak among mid-aged French adults. Our study illustrates a strong relationship between symptoms of anxiety/depression measured years before lockdown, and symptoms during lockdown averaged over the study population. Additionally, we found increased odds for symptoms of anxiety/depression during lockdown among women, persons with low household income and those who reported being lonely. Overall, our results point to the continuity in symptoms of anxiety/depression during the course of the COVID-19 pandemic, particularly during the lockdown period in the spring of 2020.

### Study strengths and limitations

Our study has several strengths including: a) a longitudinal study design with follow-up during lockdown and measures of mental health prior to the COVID-19 pandemic, b) a validated measure of symptoms of anxiety/depression, and, c) inclusion of key confounders (e.g. sex, living situation, household income). In addition, symptoms of anxiety/depression were assessed throughout lockdown, reflecting participants’ emotional well-being during this period with precision. To consider the fact that social isolation during the time of lockdown could affect the association between preexisting symptoms of anxiety/depression and anxiety/depression during lockdown, we included measures of loneliness in our analysis. Nevertheless, our study also has some limitations. The generalizability of our results is limited by the size and composition of the study sample. TEMPO includes middle-aged adults, a majority of whom work with a stable contract, and have a high household income compared to the general French population. While the sample is diverse enough to compare different groups, the role of preexisting mental health difficulties with regard to well-being during the COVID-19 pandemic may be stronger in the general population. Furthermore, the size of the study population may be limited to identify some associations, particularly interactions between prior symptoms of anxiety/depression and socioeconomic conditions and these should be tested again in larger samples. Finally, information on prior symptoms of anxiety/depression was not available for all subjects in 2018, which lead us to use information collected in 2011 and 2009, possibly inducing information bias. However, supplementary analyses showed that the timing of measurement of prior symptoms of anxiety/depression did not modify our final findings. When dichotomizing the exposure and outcome variables there is a risk of misclassification. However, the use of a validated instrument to measure symptoms of anxiety/depression accommodates this to certain extend, and the dichotomization of the variables in this study is based on previous used methods and after exploring data [40].

### Interpretation

Despite these limitations and in line with our main hypothesis, it appears that persons with symptoms of anxiety/depression 2 years preceding lockdown were more vulnerable to internalizing symptoms during COVID-19 related lockdown. This is in line with predictions related to the pandemic [10, 12, 19, 20], and highlights continuity in psychological vulnerability since affective symptoms, such as anxiety and depression, are somewhat stable over time [41–45].

As in other contexts, we found that women were approximately two times more likely to have symptoms of anxiety/depression compared to men during the COVID-19 lockdown period [11, 13, 46, 47]. Many reasons may explain why women are more vulnerable to internalizing symptoms (including reporting style, personality traits, and hormonal factors) [48–51]. In the general population, women’s greater self-regulation and sensitivity to interpersonal concerns increases their vulnerability to internalizing problems [48–51]. It is also important to note that during the COVID-19 pandemic and associated lockdown, women were largely responsible for home and childcare including school-related duties, in addition to their own work responsibilities, which may have resulted in additional sources of stress and contributed to internalizing symptoms during lockdown [52].

Furthermore, we found that people with a household income corresponding to 2500 euros or less were more likely to have symptoms of anxiety/depression during lockdown compared to those with a household income higher than 2501 euros. Many studies have demonstrated the existence of socioeconomic inequalities in mental health [53, 54], and in general, persons with financial difficulties are at increased risk of mental health problems. Not only can stress related to poverty or financial insecurity lead to mental health problems, they can also worsen preexisting mental health difficulties and inhibit recovery [55]. Previous research in France, has showed inequalities in mental health according to socio-economic position, including household income [56]. Our findings are consistent with these data and suggest the presence of socioeconomic inequalities in mental health during the period of lockdown related to COVID-19 [11].

In line with our predictions, we found that loneliness was associated with participants’ symptoms of anxiety/depression. Holmes et al. (2020) describe increased social isolation and loneliness as likely major adverse consequences of the COVID-19 pandemic and related preventive measures, which are both strongly associated with anxiety and depression. Loneliness is common among people with mental health difficulties and predicts worse recovery [23, 24, 57]. It can also be a symptom of poor mental health. There is a suggestion that persons who experience mental health difficulties have a tenfold increase in the odds of being lonely, compared to the general population [24], and up to 40% of people with depression feel lonely most of the time [58]. Cross-sectional studies conducted in the context of the COVID-19 pandemic estimated loneliness to be a key risk-factor of depression, anxiety and their comorbidity [4, 22, 25]. Additionally, our study underlines that loneliness is independently associated with symptoms of anxiety/depression during lockdown, even when controlling for prior symptoms of anxiety/depression.

### Implications

Our study contributes new insight on the continuity in symptoms of anxiety/depression prior to and during lockdown. Combining knowledge from studies estimating a higher prevalence of anxiety and depression and loneliness during lockdown with our data, our results suggests that both persons with preexisting symptoms of anxiety/depression and those feeling lonely are at higher risk of anxiety and depression during the COVID-19 pandemic and associated lockdown. Studies conducted after other infectious outbreaks have reported elevated symptoms of anxiety and depression during the time of the event and present several months after. A study conducted in Sierra Leone 1 year after the Ebola outbreak, found that symptoms of anxiety/depression were common in a national sample of Sierra Leoneans [15], as well as an elevated rate of Severe Acute Respiratory Syndrome (SARS) related PTS symptoms among hospital employees in Beijing was reported during a 3 year follow up after the outbreak [17]. In the context of the Middle East Respiratory Syndrome (MERS), researchers found that persons with a history of psychiatric illness are at risk of experiencing symptoms of anxiety and anger four to 6 months after quarantine release [16]. These findings emphasize that symptoms of anxiety and depression, as other mental health difficulties, where present in the population long time after the outbreak and the quarantine period, where the same trend could be expected in the context of the COVID-19 pandemic, and the time horizon should be considered when implementing health promotion interventions focusing on population mental health after the pandemic.

To date, there is little evidence of effective interventions to limit the mental health impact of the COVID-19 crisis. However, the post-disaster use of mental health services has been shown to be effective. A Korean study conducted after the MERS epidemic found that an active approach, including health professionals contacting persons directly (rather than giving them written information), resulted in a higher rate of mental health service utilization [59]. In Australia, the use of telehealth has been shown to be effective to treat common mental health disorders, including depression and anxiety, among adults [60, 61], and in times of social distancing tele-mental health services can contribute to reducing psychosocial distress without increasing the risk of infection [62].

Furthermore, a focus on reducing loneliness, which could effectively involve cognitive or educational components [63], might be an important target for prevention programs in order to mitigate negative mental health consequences during the pandemic as well [10, 25].

## Conclusion

This study to examines the longitudinal effect of prior symptoms of anxiety/depression in the context of the COVID-19 pandemic and associated lockdown, and gives insight into the risk of anxiety/depression during lockdown among a mid-aged French population. The present study contributes to the identification of potential groups at risk for mental health consequences during the COVID-19 pandemic, including anxiety/depression symptoms prior to lockdown, females, people with low household income and lonely subjects. Furthermore, the study shows that loneliness is independently associated with symptoms of anxious/depression, when controlling for prior anxiety/depression symptoms. As proposed by Campion et al. and the World Health Organization [19, 64], there is need for population-scale implementation of public mental health interventions, and our study suggests a focus on individuals with prior symptoms of anxiety/depression and those feeling lonely. In future research, it would be interesting to focus on the intra-individual changes in mental health before and during lockdown, and investigate related factors, as well as examine the mental health effects of the lockdown after the pandemic.

## Supplementary Information

**Additional file 1: Table S1.** Date, participant number and symptoms of anxiety/depression in the seven waves of data collection during lockdown due to the COVID-19 pandemic in March–June 2020**. Table S2.** Questions included in mental health variable.

## Data Availability

Due to the personal questions asked in this study, survey respondents were assured that the raw data will be treated confidential and will not be shared.
