# Supplementary material for: Symptoms of anxiety/depression during the COVID-19 pandemic and associated lockdown in the community: longitudinal data from the TEMPO cohort in France
Source: BMC Psychiatry. 2021 Jul 28;21:381. doi: 10.1186/s12888-021-03383-z (PMC8316881; doi:10.1186/s12888-021-03383-z)
Supplement: Supplementary file 1 — Additional file 1: Table S1. Date, participant number and symptoms of anxiety/depression in the seven waves of data collection during lockdown due to the COVID-19 pandemic in March–June 2020. Table S2. Questions included in mental health variable. [file 12888_2021_3383_MOESM1_ESM.docx]

**Supplementary material:**

| Table S1: Date, participant number and symptoms of anxiety/depression in the seven waves of data collection during lockdown due to the COVID-19 pandemic in March-June 2020 | | | | | | | | | |
| --- | --- | --- | --- | --- | --- | --- | --- | --- | --- |
|  |  | Wave 1 | Wave 2 | Wave 3 | Wave 4 | Wave 5 | Wave 6 | | Wave 7 |
| Period of data collection | | 24/03-31/03 | 31/03-07/04 | 07/04-14/04 | 14/04-21/04 | 21/04-05/05 | 05/05-19/05 | | 19/05-02/06 |
| Number of participants | | 417 | 538 | 487 | 459 | 428 | 411 | | 385 |
| Anxious/ depressive syndromes | No | 349 (83.7%) | 454 (84.4%) | 418 (85.8%) | 396 (86.3%) | 360 (84.1%) | 339 (82.5%) | | 337 (87.5%) |
|  | Yes | 68  (16.3%) | 84  (15.6%) | 69  (14.2%) | 63  (13.7%) | 68  (15.9%) | 72  (17.5%) | | 48  (12.5%) |
| National measures in and case numbers of France | | | | | | | | | |
| COVID-19 cases^†^ | | 22,853 | 30,612 | 25,030 | 17,934 | 12,155^‡^ | 7,550^‡^ | | 2,165^‡^ |
| COVID-19 related death^†^ | | 1,932 | 5,472 | 6,315 | 5,325 | 3,138^‡^ | 1,485^‡^ | | 1,331^‡^ |
| Stay-at-home orders^§^ | |  |  |  |  |  |  |  |  |
| Closure of non-essential shops^§^ | |  |  |  |  |  |  |  |  |
| Private gathering restrictions | |  |  |  |  |  |  |  |  |
| Closure of restraurants/cafes, entertainment venus, and gyms | |  |  |  |  |  |  | | |
| ^†^ France: WHO Coronavirus Disease (COVID-19) Dashboard With Vaccination Data [Internet]. [cited 2021 May 18]. Available from: <https://covid19.who.int>  ^‡^ Measures for the first week of data collection, for comparable numbers (week 17, week 19, and week 21)  ^§^ Until May, 17, 2020 | | | | | | | | | |

**Table S2: Questions included in mental health variable**

| Wave 1 | 14. Cries a lot  22. Worries about his/her future  35. Feels worthless or inferior  45. Nervous, or tense  52. Feels too guilty  91. Thinks about killing self  103. Unhappy, sad, or depressed  107. Feels that she/he can’t succeed |
| --- | --- |
| Wave 2-7 | 14. Cries a lot  22. Worries about his/her future  31. Fears doing bad  33. Feels unloved  34. Feels others are out to get him/her  35. Feels worthless or inferior  45. Nervous, or tense  52. Feels too guilty  71. Self-conscious or easily embarrassed  91. Thinks about killing self  103. Unhappy, sad, or depressed  107. Feels that she/he can’t succeed  113. Worries about relations with opposite sex |
